# Supplementary figures and images for: Changes in locus wide repression underlie the evolution of Drosophila abdominal pigmentation
Source: PLoS Genet. 2023 May 3;19(5):e1010722. doi: 10.1371/journal.pgen.1010722 (PMC10184908; doi:10.1371/journal.pgen.1010722)

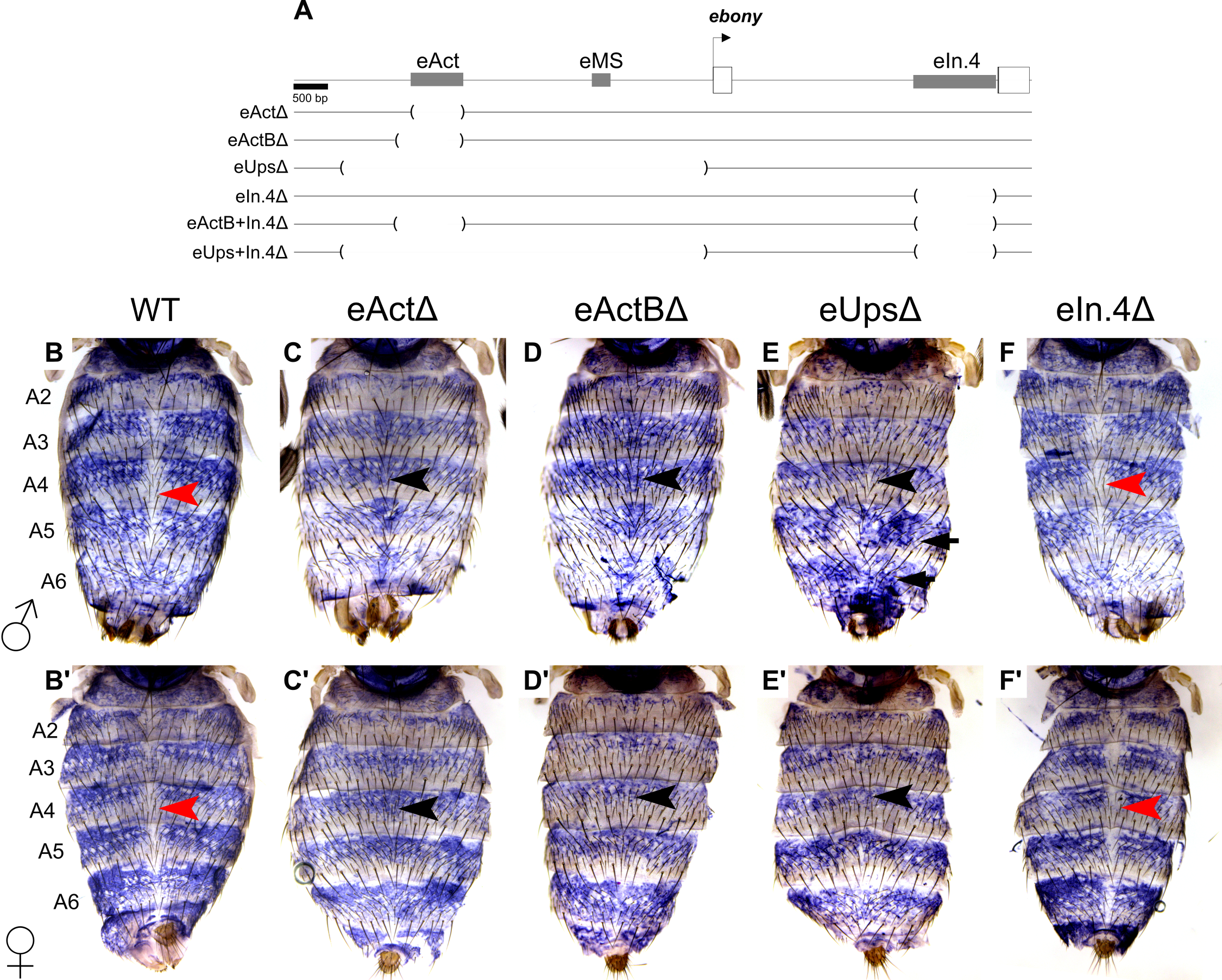

Supplement: S1 Fig — A: Gene map of the ebony locus showing the location of the deletions created to identify redundant enhancers. B-F’ ebony abdominal mRNA expression measured with in-situ hybridization in recently eclosed adults for WT, ebony null mutants, and deletion lines males and females. (TIF) [file pgen.1010722.s001.tif]

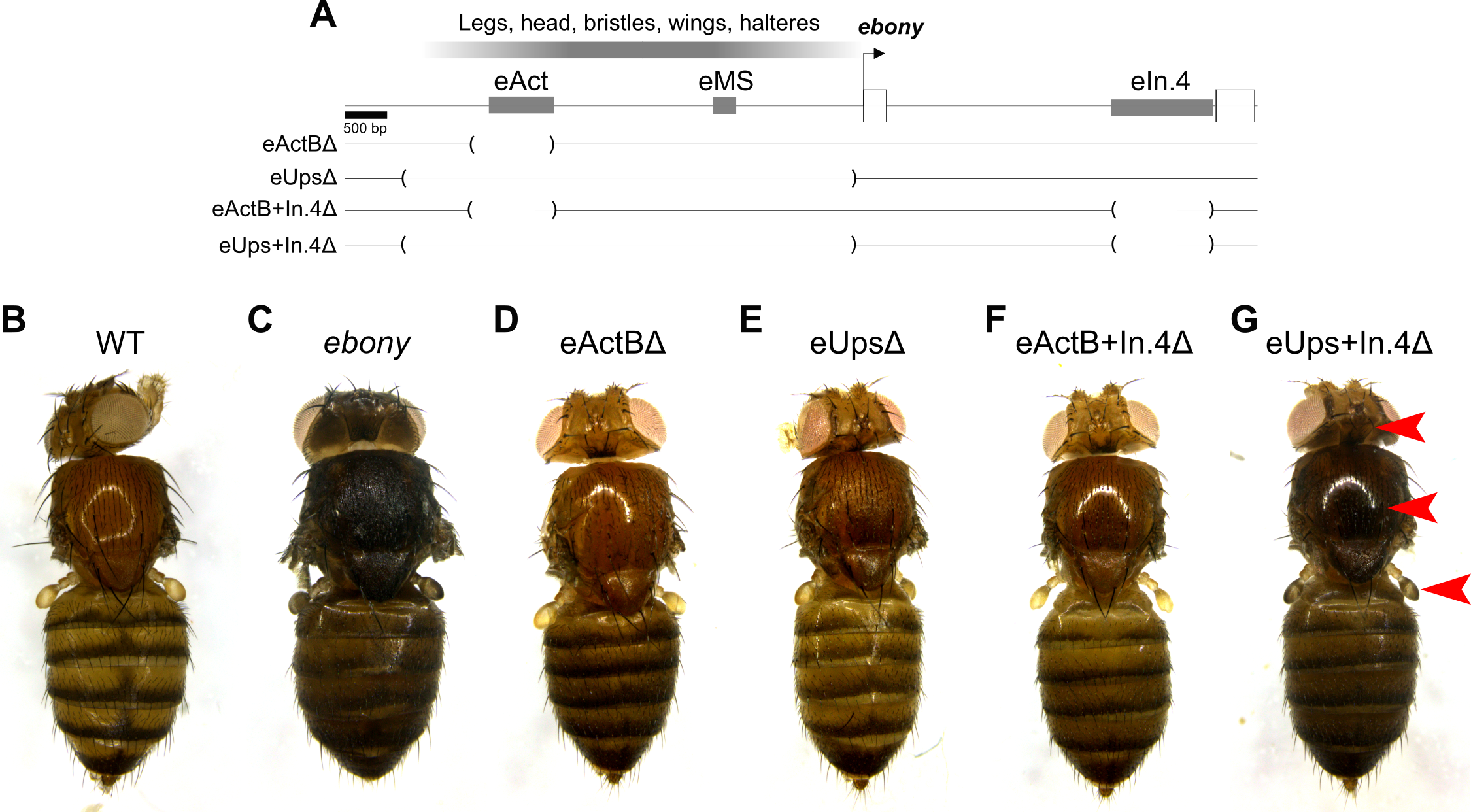

Supplement: S2 Fig — A: Gene map of the ebony locus showing the location of the deletions created to identify redundant enhancers. Previously identified tissue-specific enhancers are shown on top of the ebony upstream region (shaded rectangle). B-G: Pigmentation of different adult tissues in females from the different strains created. Red arrows show tissues, other than the abdomen, with darker pigmentation compared to the WT and more similar to ebony mutants. (TIF) [file pgen.1010722.s002.tif]

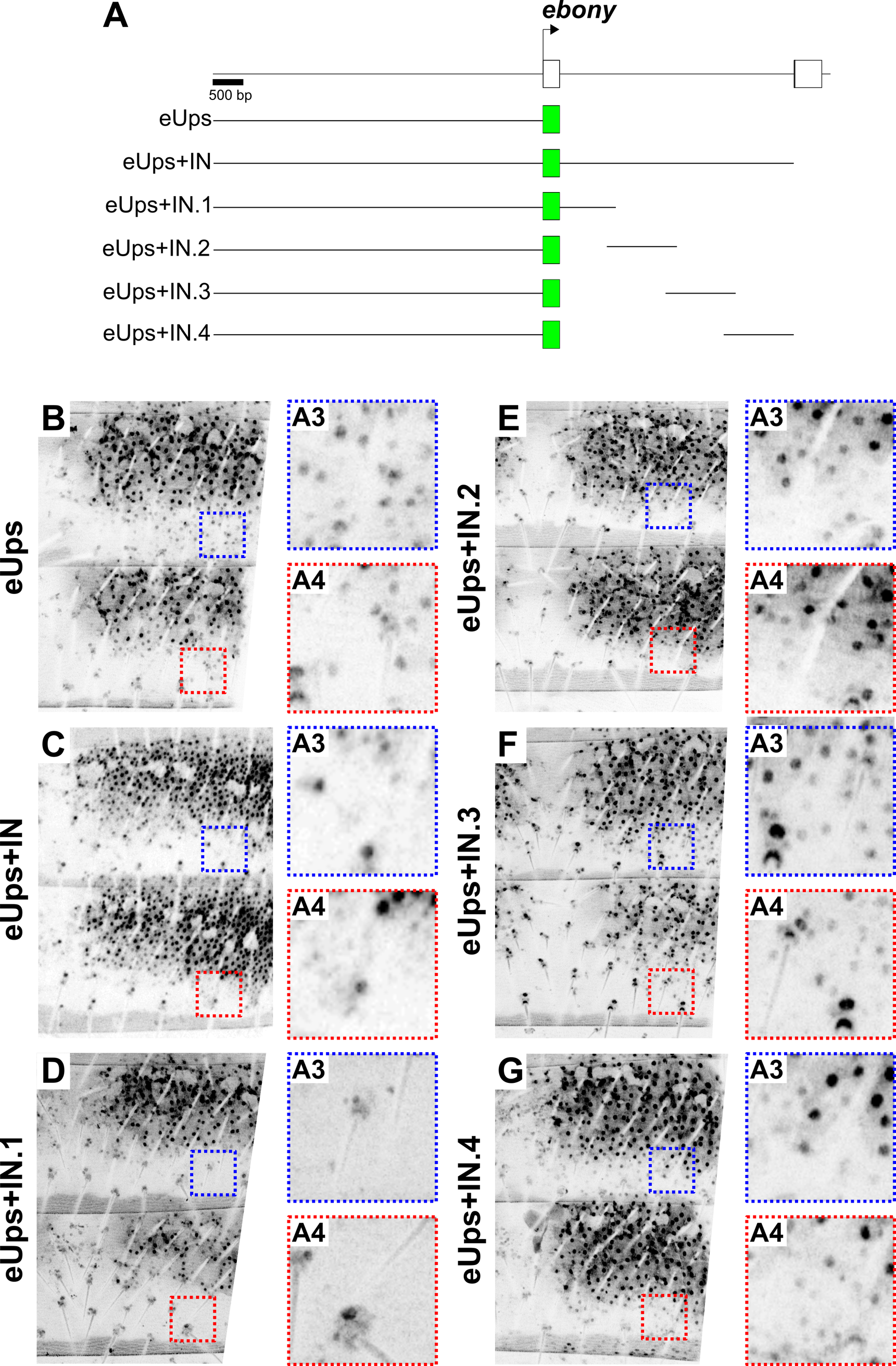

Supplement: S3 Fig — A: Gene map of the ebony locus showing the location of the reporter constructs created to identify the stripe silencer within the first intronic region. B-G: GFP expression pattern of the different transgenic reporters at 24h after eclosion. Blue and red dashed boxes show a magnification of the stripe area in A3 and A4, respectively. (TIF) [file pgen.1010722.s003.tif]

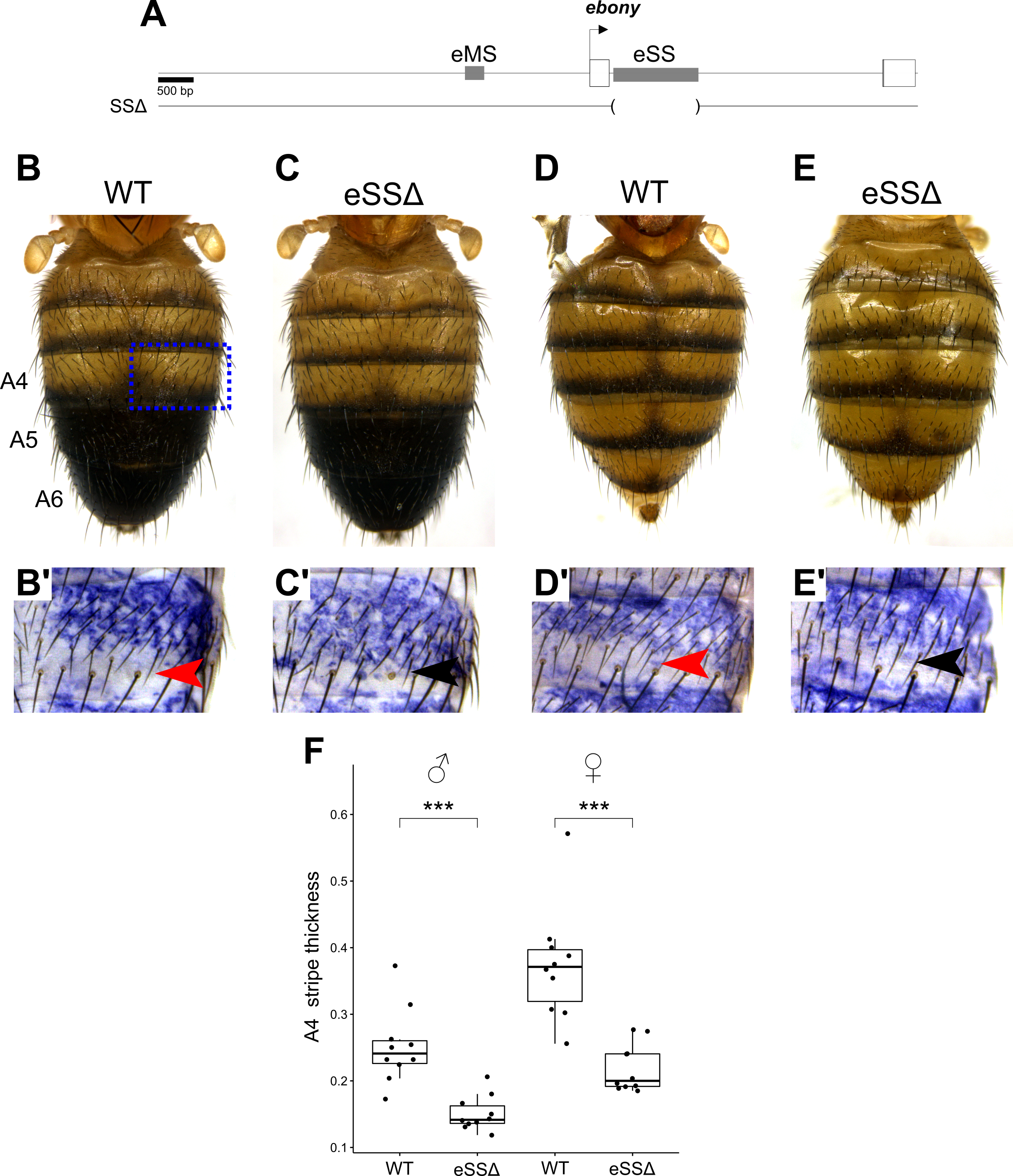

Supplement: S4 Fig — A: Gene map of the ebony locus showing the location of the deletion targeting the stripe silencer (eSSΔ). B-E: Adult pigmentation of WT and eSSΔ males and females. B-E’: In-situ hybridization detecting ebony mRNA in the A4 segment of WT and eSSΔ males and females. Red and black arrowheads indicate low and increased levels of ebony mRNA, respectively. F: Comparison of the relative thickness of the melanic stripe between WT and eSSΔ males and females (Student’s t test, *** = p < 0.0005). (TIF) [file pgen.1010722.s004.tif]

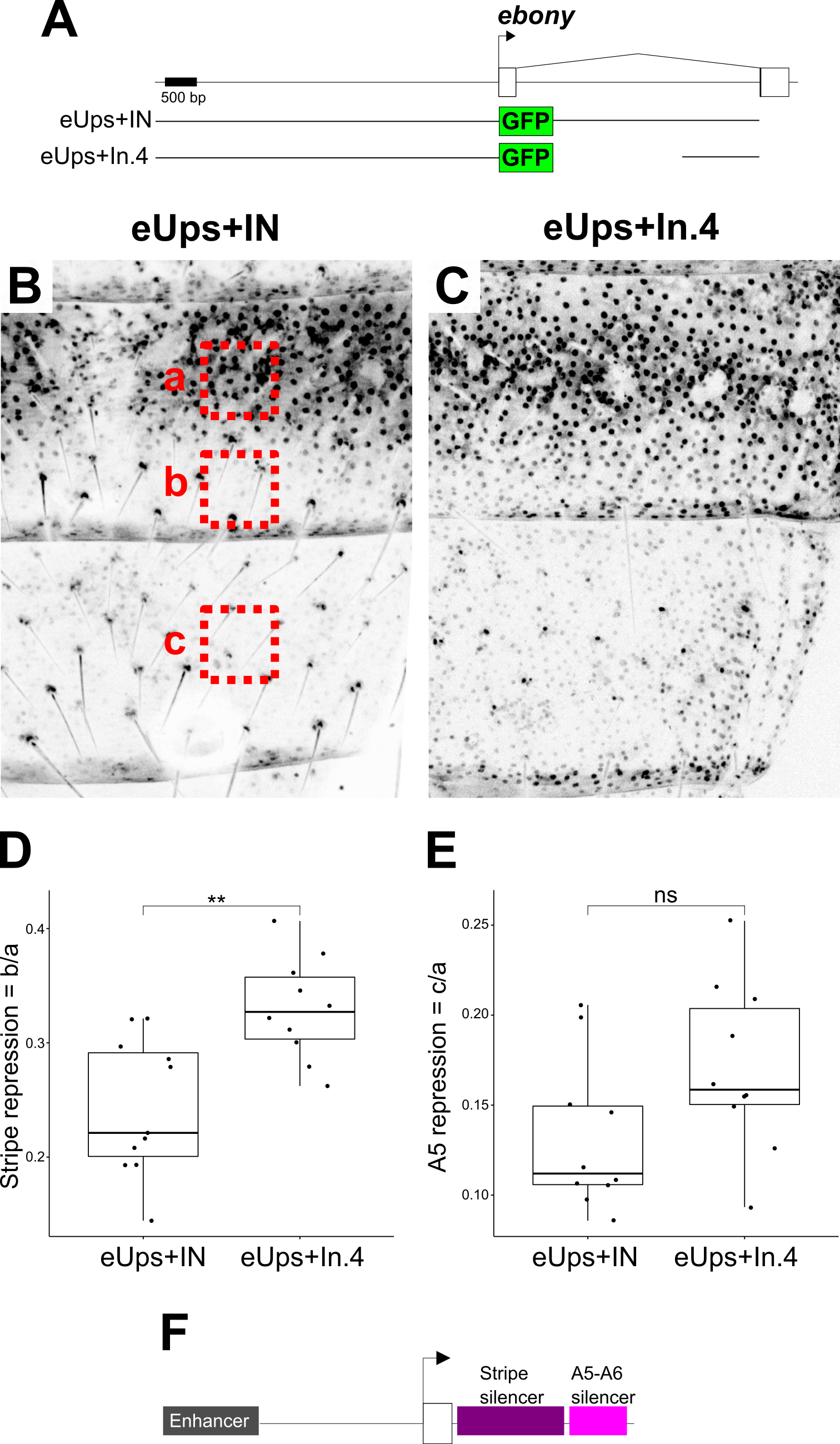

Supplement: S5 Fig — A: Gene map showing the reporter constructs created to identify the location of the D. malerkotliana male silencer within the first ebony intron. B-C: GFP expression pattern of D. malerkotliana transgenic reporter eUps+IN and eUps+In.4. Boxed regions show expression in A4 stripe region (red), and A5-A6 segments (blue and black, respectively). D: Inferred location of the D. malerkotliana intronic silencer within the first ebony intron. (TIF) [file pgen.1010722.s005.tif]

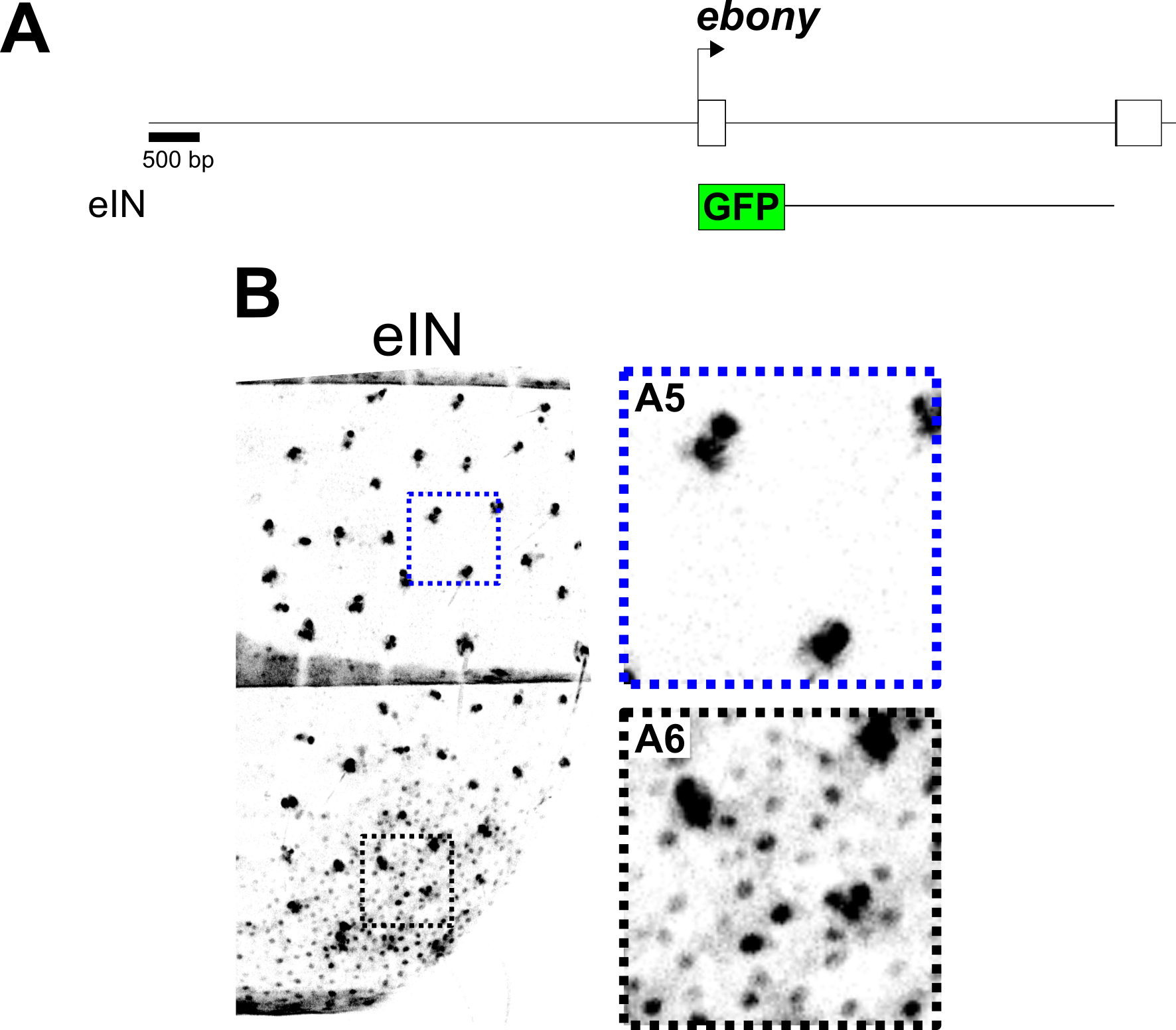

Supplement: S6 Fig — A: Gene map showing the reporter constructs created for D. pseudoobscura. B: GFP expression patterns of D. pseudoobscura transgenic reporter eIN. (TIF) [file pgen.1010722.s006.tif]

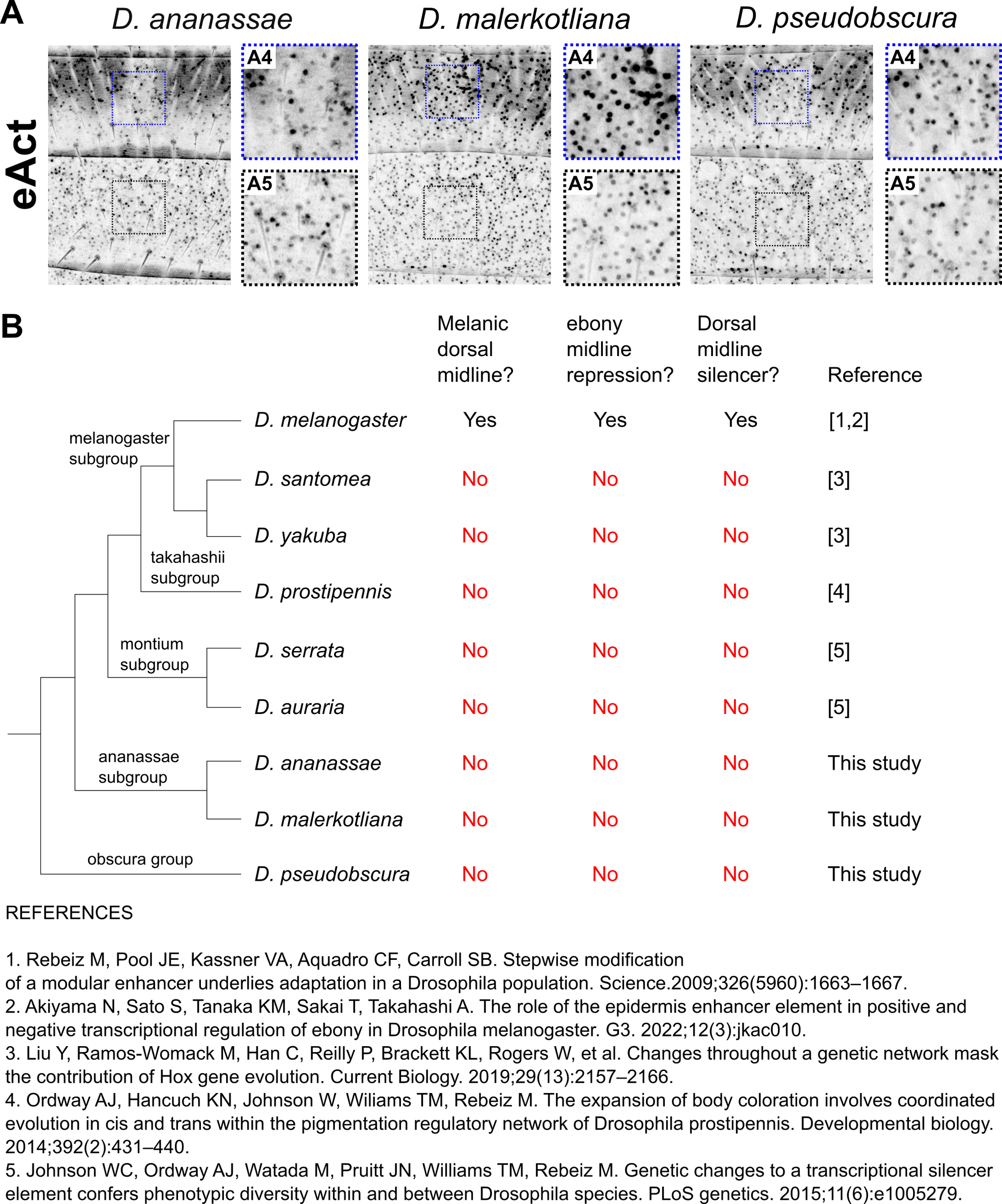

Supplement: S7 Fig — A: GFP expression patterns of the eAct transgenic reporters in the abdominal segments A4-A5. Insets show magnified regions along the midline for A4 (red square) and A5 (blue square). B: Phylogenetic distribution of the melanic dorsal midline in Drosophila species for which the expression and regulation of ebony in this area has been studied. (TIF) [file pgen.1010722.s007.tif]
