# Supplementary material for: Changes in locus wide repression underlie the evolution of Drosophila abdominal pigmentation
Source: PLoS Genet. 2023 May 3;19(5):e1010722. doi: 10.1371/journal.pgen.1010722 (PMC10184908; doi:10.1371/journal.pgen.1010722)
Supplement: Attachment — Submitted filename: REVIEW-PGEN-2022-anon.pdf [file pgen.1010722.s010.pdf]

REVIEW FOR PGENETICS-D-22-01315

DEC. 30, 2022

“Changes in global repression underlie the evolution of Drosophila abdominal pigmentation”

In this manuscript the authors present their latest work on the complex regulation of the pigmentation locus *ebony* (*e*) and its evolution across the Drosophila subgenus of Sophophora. This latest work identifies distinct enhancer and silencer activities in the *ebony* intronic region and the manner of functional interaction with previously characterized upstream regulatory elements. In general, the paper is well-written, appropriate for PLoS Genetics, and a noteworthy contribution to the fields of evolution of Drosophila pigmentation via regulatory elements and the role of silencers in gene regulatory evolution. My remaining comments are focused on places where the authors might improve on this excellent study.

The biggest weakness of this study has to do with the homology of *ebony* enhancers and silencers across Sophophora. The provenance of all the enhancers and silencers in different lineages is an issue that frequently arises in this manuscript. This makes me wish there was some treatment of homology at the level of sequence alignment in the text, figures, or supplementary material and could help to inform details in the concluding Fig. 5A. Adding more sequence details would help to tighten the two parts of the story (the specific situation in D. melanogaster and evolving DNA elements and pigmentation characters in related lineages.)

The accession numbers and sequences for all novel enhancer elements identified in this study should be provided (*e.g.*, sequence for *D. malerkotliana* enhancers).

Suggested edits:

- **TITLE:** The phrase “global repression” in the title suggests that this manuscript might describe either genome-wide profiling of transcriptional repression or perhaps repression changes in *trans*. Actually, I believe the authors are referring to the locus-wide *cis*-dominance of silencers over enhancers (at *ebony* in this case). I therefore suggest that the title be changed to something more meaningful, such as: “Evolution of Drosophila abdominal pigmentation via transcriptional silencing” or “Evolution of Drosophila abdominal pigmentation via transcriptional silencing at *ebony*”.

- **Figs. 1–4** (minor)**:** These figures depict a prominent yellow box to the right of the first and large *ebony* intronic region. I suspect that this box represents either the next exon, which begins the CDS, or else the remaining 6 exons of *ebony*, but it is never described in the text or figure legends. The color yellow also stands out and makes it seem that this is an important element that the authors forgot to mention. Perhaps some readers might even confuse it with the downstream dissected intronic element identified in this study. I would therefore change the color to make it less prominent and perhaps describe it in the first figure legend.

- **Fig 1A (!!!):** I think the female cartoon for the middle construct featuring the eAct+eMS elements driving GFP is not correctly depicted as there should be expression in the female A5 and A6 segments. (Either the middle female cartoon is incorrect or else the eMS is a silencer that is not male-specific and the intron contains a female-specific *anti-silencer.* The latter would be very interesting, but I don’t think the data support it.) In either case, I welcome the description of this element as a silencer, which is different from the original manuscript that first described it only as a male-specific repression element (Rebeiz *et al.*, 2009).

- **Summary Fig. 5:** While I like the inclusion of the concluding figure summarizing the cladistic picture of pigment evolution, I think it can be improved in several ways to make it more informative, focused, and pertinent. Details below:
    - The Fig. 5B panel is much less informative and pertinent than Fig. 5A as it doesn't convey much new information already present in other figure elements besides implying that the A5/A6 "male silencer" in *D. melanogaster* is possibly homologous to the A1-A5 silencer of *D. pseudoobscura*. For example, the cartoon of the pigmentation difference between "D. mel" and "D. pseudo" is already shown in Fig. 1B with real images, while the diagram of the evolution of the distinct silencer activities could be gotten from the Fig. 5A tree. Furthermore, the Fig. 5B right cartoon of enhancer inactivation in *D. sechelia* also doesn't convey much information, and so it doesn't seem to warrant an elaborate cartoon. I therefore suggest deleting Fig. 5B, which would also make room for the suggested edits below in Fig. 5 A.

    - In panel 5A, I would remove the *D. willistoni* lineage as this species is not treated in the text, while D. *pseudoobscura* is very much discussed and investigated and can serve as the outgroup lineage as it does in Fig. 1B. If on the other hand *D. willistoni* informs on the ancestral pigmentation characters for Sophophora, then it should be described and detailed in the text and in the figure.

    - In panel 5A, two traits are mapped onto the tree, while other, presumably lineage-specific traits are listed to the right of the tree. I would work on transforming this tree into a simple unified cladistic presentation by moving the character traits listed on the right into the tree. These could be given numbers and then listed below traits 1 and 2. Along with this change, I would also include relevant sister lineages. For example, it is interesting that *D. santomea* differs from its closely-related sister species of *D. yakuba* in this regard (I looked up pictures), so I would definitely consider adding *D. yakuba*. The evolutionary gain of the dorsal midline enhancer in *D. melanogaste*r also raises the question if this trait is shared with the sister lineages of *D. simulans/D. sechelia/D. mauritiana*, so I would add these as well (again to emphasize the recent evolutionary change if that is the case). Similarly, is the evolution of the monomorphic pan-abdominal silencer in *D. pseudoobscura* shared with *D. persimilis* (at least at the level of sequence conservation)? Last, *all characters* should be put on stem-lineages and not at nodes (neither internal nodes like 1 and 2, nor external extant nodes as is suggested by the current presentation).

      I realize some of the character traits pertain to phenotypic silencing activities while others to specific identified DNA silencing elements but I think both types of "traits" can be worked into the tree, which is after all only a model.

- **Typo on page 7, line 236:** Change "...does not recapitulate *<u>ebony</u>* the endogenous expression of *D. malerkotliana*..." to "...does not recapitulate endogenous *ebony* expression of *D. malerkotliana*..."

- **Typo in abstract:** Change "...the relative contribution of repressive transcriptional <u>silencer</u> to regulatory evolution..." to "...the relative contribution of repressive transcriptional <u>silencers</u> to regulatory evolution...". The descriptive phrase "repressive transcriptional silencing" might also be shortened to just "transcriptional silencers" to be less redundant.
