# Supplementary material for: Changes in locus wide repression underlie the evolution of Drosophila abdominal pigmentation
Source: PLoS Genet. 2023 May 3;19(5):e1010722. doi: 10.1371/journal.pgen.1010722 (PMC10184908; doi:10.1371/journal.pgen.1010722)
Supplement: Attachment — Submitted filename: Reviewers_response_final.pdf [file pgen.1010722.s011.pdf]

We thank all four reviewers for their time and effort reviewing our work. Your insightful comments were immensely helpful!

Reviewer #1: "Changes in global repression underlie the evolution of Drosophila abdominal pigmentation" by Mendez-Gonzalez and colleagues highlights something that is rather unexplored in evolution and developmental studies, that of negative regulation by silencers. Throughout the manuscript, the authors perform an elegant series of engineered deletions balanced with reporter gene constructs. Overall, I found this to be a great paper and below highlight a few points that could elevate the good work.

1) I would change the title: "global repression" from a genomics perspective could be misconstrued as genome wide. Maybe something about locus wide repression, or dominant repression.

This was suggested by other reviewers as well. We have adjusted our title to "*Changes in locus wide repression underlie the evolution of Drosophila abdominal pigmentation*"

2) Highlight the midline on the abdomen in Fig. 1A

Done

3) Figure 3 E-G, could these be quantified? It would be nice to see how these deletions affect the levels of mRNA. In other papers (Tsai et al., elife 2019), the difference between RNA levels and phenotypes has been proposed to be through buffering. So, this would be great data to highlight and would strengthen the manuscript.

We acknowledge that quantification of mRNA levels could strengthen our results. However, we opted for colorimetric in situ hybridization (a non-quantitative method that is nevertheless the gold standard for developmental studies) because it gives information about the spatial pattern of expression. We have modified the description of these results to highlight the spatial nature of this data as well as its qualitative properties (p5 L182-183; p6 L217-222 in "tracked" version).

4) Figure 4A, can you color the "Phenotype|Expression"

Done

5) I would recommend removing the svb from the figure and just save it for the discussion (or a future review), it drops abruptly and distracts a bit from the overall paper and can easily be discussed

We have completely redone figure 5, incorporating this suggestion. In the new version we tried to highlight the modes in which silencer evolution can contribute to the loss of expression.

6) It has long been discussed by developmental biologists (for example by Eric Davidson) that repression could be "dominant" which would, as the authors note, facilitate rapid evolution. I would recommend fleshing this out in the discussion because it's a great point!

We have further developed this idea in the discussion while also being mindful of the virtues of brevity with the following sentence:

Indeed, it has been posited that evolution of repressor sites in individual enhancers may present a shorter path to loss of expression than the loss of multiple activator sites, by virtue of their dominant mode of action (Preger-Ben Noon et al., 2016).

We did not find an Eric Davidson paper or book that explicitly mentioned shorter paths to enhancer inactivation via repression, but would be happy to add references if there is one suggested by the reviewer.

7) The authors could mention some of the genomics papers that explore insulators (see Barak Cohen's new preprint)

Although this is an interesting topic, we view it as tangential to the focus of the paper and would prefer to keep our discussion tightly focused on our major thesis. We do acknowledge how the ebony silencers may have insulator-like functions, but feel that the connection is a bit tenuous to speculate too wildly about at this time.

In sum, this was a great manuscript exploring an underappreciated role of negative regulation by silencing.

Reviewer #2: A major goal of evolutionary biology is to identify molecular genetic mechanisms that drive phenotypical differences between different species. In this study, Méndez-González and colleagues investigated the evolution of cis-regulatory elements (CREs) controlling ebony and abdomen pigmentation in the fly. The ebony locus was previously implicated in the evolution of abdominal pigmentation in flies by the last author (Rebeiz 2009 Science), and the present work represents a follow-up study that substantially improves our understanding of the complex cis-regulatory architecture of this locus which involves multiple independent enhancers and silencers elements. The authors further show that changes in

silencers contribute to morphological change which is the main novelty of the paper. This work is conceptually novel because most previous work on the role of non-coding DNA in evolution focused on changes in enhancers (loss or gain of function) and ignored silencer regions.

Specifically using in vivo CREs knockout experiments in D. melanogaster with observable direct phenotypic readout (pigmentation darkness of abdominal cuticula), the authors first functionally dissected regulatory regions upstream and downstream ebony promoter. These experiments identify a previously uncharacterized intronic abdomen enhancer of ebony that functions redundantly with a known upstream abdomen enhancer. Deletion of regions containing silencer regions confirms that these regions (previously characterized in transgenic reporter experiments) are required for silencing their endogenous target gene (ebony). Using interspecies transgenic reporter experiments with ebony regulatory regions from three other drosophila species the authors show that the evolution of these silencer elements underlies phenotypic differences between differently pigmented fly species. This finding – that silencer divergence underlies morphological evolution - represents a significant and novel contribution to the field. Before publication, this work will benefit from more rigorous analyses of their knockout experiments and the inclusion of motif analyses, among other comments.

Major comments

1. The authors show that both silencers (eMS and eSS) are sufficient to repress ebony expression in their reporter assay. Surprisingly, however, only the deletion of eSS is sufficient to alter the pigmentation phenotype. How do the authors reconcile this discrepancy? The authors cite that for eMS it could be due to the compensatory expression of yellow and tan genes, but presumably these genes are also expressed in the stripe areas. This should be further discussed. Also the data showing the effects of eSS deletion should be in the main figure 2 together with eMS deletion data.

We have expanded the explanation for this contrasting result. While pure speculation, we propose that the level of expression of yellow and tan may be different across the abdomen. Perhaps higher expression in A5-A6 which counteracts the effects of ebony de-repression after deleting eMS (p7 L234-240 in "tracked" version).

2. The authors make claims that deletions of silencers, for instance, increase ebony expression, as measured by ISH, but there is no formal quantification supporting this. While

the images are helpful, the authors should formally quantify these differences and whether these differences are significant between genotypes to make such conclusions.

We acknowledge that quantification of mRNA levels could strengthen our results. However, we opted for in situ hybridization (a non-quantitative method) because it gives information about the spatial pattern of expression, which we deem more valuable when analyzing silencers that have spatial domains of activity. We have modified the description of these results to highlight the spatial information as well as its qualitative nature (p5 L182-183; p6 L217-222 in "tracked" version).

3. The scale/units for pigmentation darkness and stripe thickness need to be clarified. For example, in Fig 2, it says 'relative darkness,' but there is no description of how this was normalized to WT levels.

We agree that the way we quantified pigmentation was not well explained. Likewise, the definition of relative darkness may be confusing. We have renamed this as '% of Darkness' and expanded the explanation of how this was measured in the methods section. We also added an explanation of how stripe thickness was measured (P4 L121-129).

4. How do these putative silencers repress ebony expression on a mechanistic level? This remains largely unanswered. It is surprising that the authors don't discuss this. While looking at mechanistic details of silencing such as histone modifications (deacetylation or depositing repressor marks) is beyond this paper's scope, the authors could at least consider examining TF motifs among the different silencer sequences and checking if there are gains/losses of putative repressors between different species.

We agree that understanding the mechanisms of ebony silencers is crucial to develop further hypothesis about the way they influence regulatory evolution. We are currently working on dissecting these silencers to narrowly identify the most important binding sites for silencer activity. We thus wish to hold back on less reliable speculation based upon binding site predictions in order to provide a more thorough and rigorous mechanistic assessment.

5. The authors show a loss of GFP reporter expression in the presence of the eMS silencer element from D. pseudobscura. Though beyond the scope of this paper, in my opinion, the most exciting experiment would be to test whether this D. pseudobscura eMS element is sufficient to repress ebony expression when swapped into the native D. melanogaster locus. To be clear, I'm not asking for the authors to do this, but if they already have this data, this manuscript would benefit from its inclusion.

We agree that performing CRE swaps between species is the next and most exciting experiment. We are hoping to perform this kind of experiment in the next stage of the project and taking advantage of the deletion lines we already created.

6. The authors cite relevant work from Gisselbrecht et al. (ref 13) that demonstrated silencers might also function as enhancers in other cellular contexts. Is there any evidence supporting this for the silencers tested in this study? Do they work as enhancers in different tissues or at diferent timepoints? Perhaps it could be examined if there are overlapping characterized Gal4 lines from Janelia or Vienna collection.

So far, the only evidence for overlap with an enhancer is the silencer that we found in the *eAct* element, but this is a very special case. Currently, we view simple overlap of activities to be a poor measure of the phenomenon described by Gisselbrecht. In our view, the best case scenario would be to demonstrate that binding site-sized stretches of DNA required for enhancer activity also influence silencer activity or vice-versa. Ongoing experiments involving mutational scanning of the ebony activator region and male silencer elements may reveal such a relationship. Though outside of the scope of the paper, it is something that we are looking for with the appropriate experimental design.

Minor comments

1. Is Fig 4C-K only males? This is explicitly outlined in other figures, but not this one. Please include.

These images only show males. We have added this information to both the figure and figure legend.

2. The use of different names for the same enhancer/silencer element (i.e., eMS and A5/A6 silencer) within the text and figures makes it very difficult to follow. The authors should be consistent in their regulatory element nomenclature.

We apologize for the confusing nomenclature, we have fixed this and used only eAct, eMS and In.4.

3. Likewise, one deletion is named IN.4 and another In.4 for different enhancers. This is confusing and the authors should change these names to be more intuitive for the reader.

See above

Reviewer #3: "Changes in global repression underlie the evolution of Drosophila abdominal pigmentation" In this manuscript the authors present their latest work on the complex regulation of the pigmentation locus ebony (e) and its evolution across the Drosophila subgenus of Sophophora. This latest work identifies distinct enhancer and silencer activities in the ebony intronic region and the manner of functional interaction with previously characterized upstream regulatory elements. In general, the paper is well-written, appropriate for PLoS Genetics, and a noteworthy contribution to the fields of evolution of Drosophila pigmentation via regulatory elements and the role of silencers in gene regulatory evolution. My remaining comments are focused on places where the authors might improve on this excellent study.

The biggest weakness of this study has to do with the homology of ebony enhancers and silencers across Sophophora. The provenance of all the enhancers and silencers in different lineages is an issue that frequently arises in this manuscript. This makes me wish there was some treatment of homology at the level of sequence alignment in the text, figures, or supplementary material and could help to inform details in the concluding Fig. 5A. Adding more sequence details would help to tighten the two parts of the story (the specific situation in D. melanogaster and evolving DNA elements and pigmentation characters in related lineages.) The accession numbers and sequences for all novel enhancer elements identified in this study should be provided (e.g., sequence for D. malerkotliana enhancers).

To address the largest weakness noted above, we include a graphical alignment of the genome sequences around ebony, which clearly establishes the orthology relationships of the different sequence regions tested. The alignment was produced using the GenePalette program (*Smith et al., Dev Biol 2017*), and shows the positions of unique 12mer sequences that are co-linear in the locus. While sequences of the elements themselves do not contain any of these conserved landmarks, one can see that each element is flanked by co-linear landmarks, which justifies our usage of these elements. The absence of landmarks does not mean that the sequences are unalignable – just that large blocks of unique conserved 12mers are not in these elements. For all of our transgenic reporters, we examined larger regions compared to the minimal regions defined in D. melanogaster, in this way we defined CREs based on the expression observed.

To increase replicability, we include Supplemental Table 2, containing all source genome sequences we used in this study.

Suggested edits:

TITLE: The phrase "global repression" in the title suggests that this manuscript might describe either genome-wide profiling of transcriptional repression or perhaps repression changes in

trans. Actually, I believe the authors are referring to the locus-wide cis-dominance of silencers over enhancers (at ebony in this case). I therefore suggest that the title be changed to something more meaningful, such as: "Evolution of Drosophila abdominal pigmentation via transcriptional silencing" or "Evolution of Drosophila abdominal pigmentation via transcriptional silencing at ebony".

This was suggested by other reviewers as well. We have changed it to "Changes in locus wide repression underlie the evolution of Drosophila abdominal pigmentation"

Figs. 1–4 (minor): These figures depict a prominent yellow box to the right of the first and large ebony intronic region. I suspect that this box represents either the next exon, which begins the CDS, or else the remaining 6 exons of ebony, but it is never described in the text or figure legends. The color yellow also stands out and makes it seem that this is an important element that the authors forgot to mention. Perhaps some readers might even confuse it with the downstream dissected intronic element identified in this study. I would therefore change the color to make it less prominent and perhaps describe it in the first figure legend.

That's correct, the yellow box represents the first coding exon. We have changed the color of this box to make it less conspicuous and describe what it represents in the legend.

Fig 1A (!!!): I think the female cartoon for the middle construct featuring the eAct+eMS elements driving GFP is not correctly depicted as there should be expression in the female A5 and A6 segments. (Either the middle female cartoon is incorrect or else the eMS is a silencer that is not male-specific and the intron contains a female-specific anti-silencer. The latter would be very interesting, but I don't think the data support it.) In either case, I welcome the description of this element as a silencer, which is different from the original manuscript that first described it only as a male-specific repression element (Rebeiz et al., 2009).

Thank you for pointing out this, as found in Rebeiz 2009, eMS is only active in males, we have fixed this mistake and depicted the correct GFP expression in the female cartoon.

Summary Fig. 5: While I like the inclusion of the concluding figure summarizing the cladistic picture of pigment evolution, I think it can be improved in several ways to make it more informative, focused, and pertinent. Details below:

o The Fig. 5B panel is much less informative and pertinent than Fig. 5A as it doesn't convey much new information already present in other figure elements besides implying that the A5/A6 "male silencer" in D. melanogaster is possibly homologous to the A1-A5 silencer of D. pseudoobscura. For example, the cartoon of the pigmentation difference between "D. mel"

and "D. pseudo" is already shown in Fig. 1B with real images, while the diagram of the evolution of the distinct silencer activities could be gotten from the Fig. 5A tree. Furthermore, the Fig. 5B right cartoon of enhancer inactivation in D. sechelia also doesn't convey much information, and so it doesn't seem to warrant an elaborate cartoon. I therefore suggest deleting Fig. 5B, which would also make room for the suggested edits below in Fig. 5 A.

We have completely restructured Fig. 5. We removed panel B and created a simplified cartoon representing the different regulatory changes involving enhancers and silencers that can lead to a similar result, loss of expression.

In panel 5A, I would remove the D. willistoni lineage as this species is not treated in the text, while D. pseudoobscura is very much discussed and investigated and can serve as the outgroup lineage as it does in Fig. 1B. If on the other hand D. willistoni informs on the ancestral pigmentation characters for Sophophora, then it should be described and detailed in the text and in the figure.

In panel 5A, two traits are mapped onto the tree, while other, presumably lineage-specific traits are listed to the right of the tree. I would work on transforming this tree into a simple unified cladistic presentation by moving the character traits listed on the right into the tree. These could be given numbers and then listed below traits 1 and 2. Along with this change, I would also include relevant sister lineages. For example, it is interesting that D. santomea differs from its closely-related sister species of D. yakuba in this regard (I looked up pictures), so I would definitely consider adding D. yakuba. The evolutionary gain of the dorsal midline enhancer in D. melanogaster also raises the question if this trait is shared with the sister lineages of D. simulans/D. sechelia/D. mauritiana, so I would add these as well (again to emphasize the recent evolutionary change if that is the case). Similarly, is the evolution of the monomorphic panabdominal silencer in D. pseudoobscura shared with D. persimilis (at least at the level of sequence conservation)? Last, all characters should be put on stem-lineages and not at nodes (neither internal nodes like 1 and 2, nor external extant nodes as is suggested by the current presentation). I realize some of the character traits pertain to phenotypic silencing activities while others to specific identified DNA silencing elements but I think both types of "traits" can be worked into the tree, which is after all only a model.

We decided to simplify this summary and instead use a table format. Our goal is to highlight that in all instances of spatial regulatory evolution that have been documented so far involved changes in the function of silencers at ebony. By using a table we also were able to describe the pigmentation change as well as include the reference.

Typo on page 7, line 236: Change "...does not recapitulate ebony the endogenous expression of D. malerkotliana..." to "...does not recapitulate endogenous ebony expression of D. malerkotliana..."

Done

Typo in abstract: Change "...the relative contribution of repressive transcriptional silencer to regulatory evolution..." to "...the relative contribution of repressive transcriptional silencers to regulatory evolution...". The descriptive phrase "repressive transcriptional silencing" might also be shortened to just "transcriptional silencers" to be less redundant.

Done

Reviewer #4: This is an interesting study and a well-written manuscript presenting experimental data, appropriately collected and analysed, that significantly contributes to the mechanisms of transcriptional regulation and its putative roles in morphological evolution. There are no major flaws to report or fundamental new experiments to carry out but there is room for improving the manuscript. Below is provided a list of general and particular aspects that should be clarified and suggestions that might improve the understanding of the data and of its conclusions.

General comments:

Not enough detail in the methods that complicates the interpretation of the results. Please do have an overall revision with an eye to missing information necessary for the untrained reader. Some examples, below.

4) **There is not sufficient detail regarding the quantification methods applied for the three different cases (pigmentation, in situ, and GFP reporters). For instance, it is not obvious from the figures that there was 'higher ebony mRNA expression compared to WT as measured by in situ hybridization' (Fig 3D-E), (lines 201-202).**

We acknowledge that the way we quantified pigmentation was not well explained. Likewise, the definition of relative darkness may be confusing. We have renamed this as '% of Darkness' and expanded the explanation of how this was measured in the methods section. We also added an explanation of how stripe thickness was measured. Along these lines, we acknowledge that quantification of mRNA levels could strengthen our results. However, we opted for in situ hybridization (a non-quantitative method) because it gives information about the spatial pattern of expression. We have modified the description of these results to highlight

the spatial information as well as its qualitative nature (p5 L182-183; p6 L217-222 in "tracked" version).

2) Were these differences quantified, as done for darkness (panels H-I)?

See above

3) In general, it would be nice to see some of the quantitative measures done for the GFP reporter experiments (as described in lines 143-149), especially given that some of the differences mentioned are not so obvious from the chosen images (for instance in Fig 4).

We have included these results in Fig. 4 as well as in the text.

4) The authors could also be more concrete on how the define the line that separates the pigmentated from the non-pigmented part of the segment. This is important for the overall quantification analyses, as it is, more specifically, for the same of Fig S4 (panel F) where the relative thickness is presented.

See response for comment 1. We defined the non-pigmented part of the segment as the anterior-most third of the tergite.

5) It is also not clear how the authors define homology between segments in the analyses of the different species (contained in Fig. 4 A-B). Is there synteny between all species that includes the upstream gene CG5892? That would give a better sense of the orthology between the intergenic region that sometimes differs in size considerably between species and makes the task particularly risky. How were the CREs defined and how reliable are they in this context?

We agree that defining orthology of intergenic regions is a difficult task. The alignment in Fig 4 A shows blocks of synteny in the upstream and intronic ebony region up to the putative location of CG5892. For all of our transgenic reporters we examined larger regions compared to the minimal regions defined in D. melanogaster, in this way we defined CREs based on the expression observed.

6) Another example of the necessity for more detailed methods relates to the results presented in Fig 3. It is unclear what is the reference against which relative intensity is being measured. When the authors claim that 'Red and black arrowheads indicate low and increased levels of ebony mRNA', is it relative to what? On the same Fig 3, it is not clear if panels H and I quantify pigmentation (panels B-D) or expression via in situ hybridization

(panels E-G). Moreover, the H-I quantification misses segment A4, which appears to be most informative one, considering the adult's final pigmentation phenotypes (panels B-D). This becomes more surprising if one considers that in Fig 2 the authors focus solely on that segment A4!

See response to comment 1. We did not quantify A4 because eMS only represses GFP in segments A5-A6 (as found using GFP reporters in Rebeiz 2009). The darker A4 pigmentation observed in panel D is the result of deleting the eAct enhancer and it is showed in Fig. 2.

The last comment also relates to the fact that there seem to be some inconsistencies in the way the data is presented. There is for instance, a discrepancy between the segments being considered in each of the results section (sometimes it is A4 and A5, and sometimes A5 and A6), which makes hard to relate the results and weakens some of the conclusions drawn across experiments. Another instance of inconsistency is the use of males and/or females. It is not always clear in each experiment, if one or the other, or both sexes have been considered. Please make this explicit in figure legends, and in the text be clearer on why a given option was made.

We apologize the inconsistency, except for figure 2 we only focused on males since they show more variable pigmentation phenotypes. We have added this information to figures and legends.

We focused on different segments in each figure based on where each CRE analyzed is active (which is based on the knowledge from GFP reporters from Rebeiz 2009 and summarized in Fig. 1A).

Minor Suggestions:
It would have been interesting to see the phenotypes against the ebony mutants in the in situ hybridization results (Fig. 3 and Fig S1). It is not a necessary experiment, but if that data would be available, it could be an interesting comparison to add.

Unfortunately, we do not have these data. The ebony loss of function mutant was created by inserting a large plasmid into the first coding exon, thus while the levels of expression might not be affected, it is predicted that these transcripts will not produce a functional protein.

Finally, the discussion could benefit from further developing the global versus local effects, particularly if the title includes the term 'global repression'. Not excluding what was said

before, maybe the title itself can be revised to a more general conclusion, for example: 'The role of transcriptional repression in the evolution of Drosophila abdominal pigmentation.'

This was suggested by other reviewers as well. We have changed it to "Changes in locus wide repression underlie the evolution of Drosophila abdominal pigmentation"

Other comments:

- **Figure 1A: The authors could consider adding the names of the enhancer (eAct) and the silencers (eMS and eSS) to the schemes so that the figure matches better the text description in lines 37-44.**

Fixed

- **Figures 1A, 2A, 2J, and 2K have very low resolution.**

Fixed

- **Figure 1 legend, panel A: please define what the white and yellow boxes represent in the gene model and what are the red arrows pointing to.**

We have changed the color of the yellow box to make it less conspicuous and added a description of what these boxes represent in the figure legend.

- **Figure 4, panel A: is hard to understand**

We have modified the legend explaining this panel

- **Figure 4, legend: add at the end of the last sentence 'for D. ananassae, D. melanogaster, and D. pseudoobscura, respectively'.**

Fixed

- **Line 90: D. melanogaster should be in itallics.**

Fixed

- **Line 95: is missing a reference**

The website containing the protocol has been added

• Lines 120-123: it is unclear if the pigmentation was measured in the entire abdomen or only in the anterior-most part.

We have expanded the explanation in the methods section and renamed 'relative darkness' as 'percentage of darkness'.

• Line 145-147: It is unclear how the measurements described in this section relate to the squares drawn in Figure 4C-K. Also, in this same section, please confirm that the description of how the stripe silencing activity is measured is correct.

We apologize for the omission of these results. We have included the GFP quantification in the insets of Fig. 4. We also modifed Fig. S5 to better explain how stripe silencing was quantified for the malerkoltiana reporters.

• Line 148: please correct 'A45 segment'.

Fixed

• Line 159-160: the statement that 'Deletion of eAct did not affect the abdominal pigmentation intensity (Fig 2D-D', J-K)' is confusing given that the corresponding images show no midline pigmentation.

We have modified this sentence to specify that this deletion does not affect percentage of darkness (which was measured laterally to the midline) although it erases the dark midline stripe. We added a square in Fig. 2B to show the region used for this measurement.

• Line 188: wings are not shown in any figure but are referred to in the results

We have removed wings from this sentence.

• Lines 201-202: please confirm that the figure panels cited in are correct.

Fixed

• Lines 204-205: The proposed hypothesis of why there are no phenotypic effects observed (and what is the data behind it) could be better explained

We have modified the description of the phenotypic effects of deleting silencers and explain expanded our explanations to why no phenotypic effects were observed after deleting eMS.

• **Line 209: delete extra space before ‘1.5 kb‘**

Fixed

• **Line 201: please confirm that the cited figure panels are correct**

Fixed

• **Line 236: remove ‘the’**

Fixed

• **Line 242-243 : please confirm that the cited figure panels are correct**

Fixed

• **Lines 245-247: it is unclear what is the rationale behind this hypothesis. Please explain**

We have modified this sentence

• **Line 319: delete extra space**

Fixed

• **Line 394: remove dot after ‘females’**

Fixed
